# Breast cancer mortality trends in two areas of the province of Florence, Italy, where screening programmes started in the 1970s and 1990s

**DOI:** 10.1038/sj.bjc.6601744

**Published:** 2004-03-23

**Authors:** G Gorini, M Zappa, G Miccinesi, E Paci, A Seniori Costantini

**Affiliations:** 1 Unit of Occupational and Environmental Epidemiology, Centre for Study and Prevention of Cancer (CSPO), Via di S. Salvi, Florence 12-50135, Italy; 2 Unit of Clinical and Descriptive Epidemiology, Centre for Study and Prevention of Cancer (CSPO), Via di S. Salvi, Florence 12-50135, Italy

**Keywords:** screening, breast, early diagnosis, mortality

## Abstract

We compared breast cancer mortality rates in the period 1985–2000 in two areas of the province of Florence, Italy, where breast cancer screening programmes started in the 1970s (early screening (ES) area) and in 1990s (late screening (LS) area). The overall age-standardised mortality decreased in the whole period by 40.9% in the ES area (*P*<0.001), and by 11.3% in the LS area (*P*=0.030). Significant decreases in the ES area were detected in groups aged 45–54 years (61.1%; *P*= 0.018) and 65–74 years (44.7%; *P*= 0.049), whereas in the LS area no significant decrease was detected in any age group. The relatively low compliance in the first years of the programme in both areas, and the long enrolment period in the LS area could have reduced the effect on mortality. Our findings suggest that the drop in mortality in the ES area (41%) could be explained by both service screening and better care. The slight decrease in mortality in the LS area (11%) could be mainly due to better care. A reduction of about 30% is attributable to screening in the ES area over the period 1985–2000.

Mammographic screening has been shown to reduce breast cancer mortality rates (Vainio and Bianchini, 2002). Several studies in northern European countries (Hermon and Beral, 1996; Hakama *et al*, 1997; Van den Akker-van Marle *et al*, 1999; Blanks *et al*, 2000; Jonsson *et al*, 2001; Botha *et al*, 2003; Otto *et al*, 2003; Tabar *et al*, 2003) have reported a decrease in breast cancer mortality where nationwide screening programmes have been implemented. There has, however, been considerable debate concerning the relative contributions of screening and of improved therapy to the observed trends (Carnon *et al*, 1996; Blanks *et al*, 2000; Peto *et al*, 2000).

At the beginning of the 1970s in 23 rural municipalities of the province of Florence, Italy (early screening area (ES area) among about 70 000 resident women aged 25 or over, 18% of women of the province, based on 1991 Census), a mammographic screening programme was started; until 1989, women in the 40–69 year age group were invited, whereas from that year onwards the target population was restricted to age group 50–69 years. The number of mammograms performed in the ES area was 8000–9000 per year. The efficacy of the programme on breast cancer mortality in the ES area has been evaluated for the period 1977–1984 by means of case–control studies (Palli *et al*, 1986, 1989), and by using early indicators of efficacy (Paci *et al*, 1990). After 1990, when the screening for women aged 50–69 years started in the rest of the province, including the city of Florence (late screening area: LS area), the number of mammograms in the whole province increased to 28 000–29 000 per year (Barchielli *et al*, 1999).

In this study, we have compared breast cancer mortality rates in the period 1985–2000 in the ES and LS areas.

## MATERIALS AND METHODS

### Sources of data

Data on breast cancer mortality for the period 1985–2000 were obtained from the Tuscan Regional Mortality Register (Chellini *et al*, 2002), which collects death certificates of the residents of Tuscany. Incident breast cancer cases for the period 1985–1999 were obtained from the Tuscan Cancer Register, a member of the International Association of Cancer Registries (Parkin *et al*, 2002).

### Statistical analysis

Adjusted mortality and incidence rates using direct standardisation to the European Standard Population, and age-specific (35–44, 45–54, 55–64, 65–74, 75–84 and ⩾85 years) incidence and mortality rates with 95% confidence intervals (95% CI) were calculated.

In order to calculate the estimated percent change (EPC) in rates for the whole period of observation, trends in mortality and incidence based on annual data were examined using a log-linear regression model, and the year of death as a continuous variable.

## RESULTS

**Figure 1:**
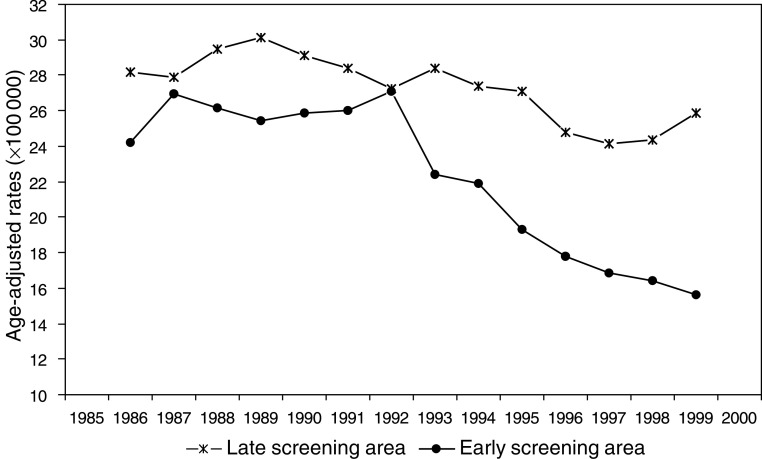
Age-adjusted (European population) breast cancer mortality rates in the ES area and in the LS area. Period 1985–2000, 3-year moving averages.

**Figure 2:**
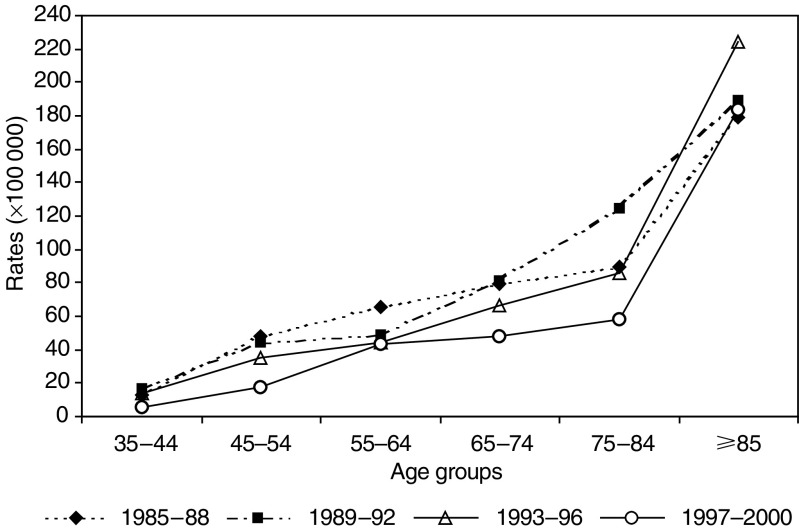
Age-specific mortality rates in the period 1985–2000 in the ES area of the province of Florence, Italy.

**Figure 3:**
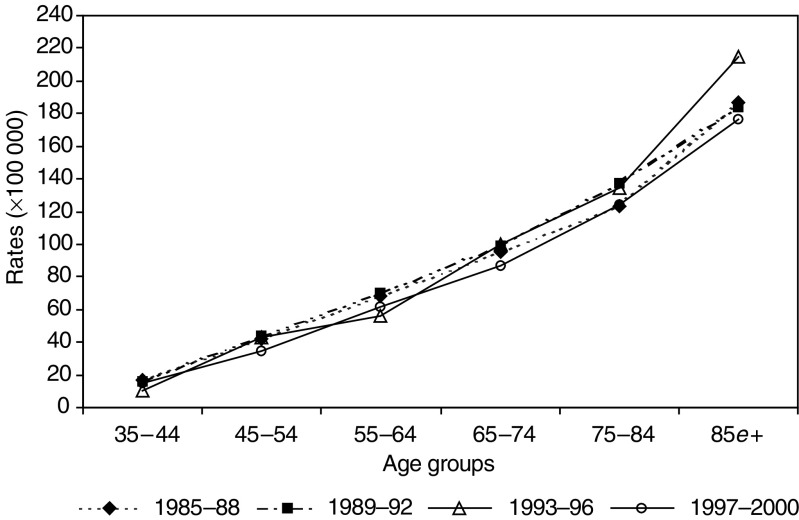
Age-specific mortality rates in the period 1985–2000 in the LS area of the province of Florence, Italy.

The age-adjusted mortality rate in the ES area (Figure 1

) was 22.4 per 100 000 women in 1985 (95% CI: 13.6–31.2; 27 deaths), 31.8 in 1991 (95% CI: 21.0–42.6; 38 deaths) and then decreased to 10.9 in 2000 (95% CI: 5.4–16.5; 19 deaths). In the LS area, the corresponding age-standardised mortality rates were more stable with only a slight decrease over time: in 1985 30.1 per 100 000 women (95% CI: 25.5–34.7; 186 deaths); 27.7 in 2000 (95% CI: 23.3–32.1; 190 deaths). In the ES area, age-specific mortality rates decreased steadily from 1985 to 1986 until 1999–2000 in all age groups, except for 85+ age group (Figure 2

). The age-specific rates in the LS area were more stable (Figure 3

).

In the ES area, incidence was 76.9 per 100 000 women in 1985 (95% CI: 59.6–94.1), 96.7 in 1989 (95% CI: 77.6–115.8), 62.5 in 1993 (95% CI: 47.9–77.1) and 110.6 in 1999 (95% CI: 90.8–130.4). In the LS area, incidence rates were higher: 91.1 per 100 000 women in 1985 (95% CI: 82.6–99.6), 112.1 in 1993 (95% CI: 102.9–121.4) and 113.9 in 1999 (95% CI: 104.5–123.4).

In addition to visual inspection, we measured EPCs in rates over the whole period (Table 1

**Table 1:** Estimated percentage change (EPC) and 95% confidence intervals (95% CI) of mortality rates (1985–2000) and incidence rates (1985–1999) in Early Screening area and in Late Screening area

	**Mortality (1985–2000)**				**Incidence (1985–1999)**			
	**ES area**		**LS area**		**ES area**		**LS area**	
**Age (years)**	**EPC (%)**	**95% CI**	**EPC (%)**	**95% CI**	**EPC (%)**	**95% CI**	**PC (%)**	**95% CI**
35–44	−53.4	−87.4; +72.9	−21.7	−55.2; +36.9	+12.6	−33.8; +91.6	+17.0	−6.1; +45.8
45–54	−61.1	−82.2; −14.9	−18.4	−41.3; +13.5	+31.2	−8.3; +87.9	+7.1	−5.6; +21.5
55–64	−41.6	−69.6; +12.5	−15.7	−35.3; +9.9	+5.5	−24.8; +48.2	+21.0	+4.8; +39.8
65–74	−44.7	−69.8; −0.3	−10.8	−29.7; +13.3	−2.9	−31.5; +37.8	+8.4	−6.0; +24.9
75–84	−45.2	−70.9; +3.5	−5.1	−25.5; +20.9	+28.2	−14.6; +92.4	+15.9	−2.1; +37.3
⩾85	+12.0	−46.2; +133.2	−5.7	−31.7; +30.0	+6.1	−47.5; +114.6	+45.1	+8.5; +94.1
								
35–85+	−36.0	−52.1; −14.4	−3.0	−13.8; +9.2	+13.3	−4.0; +33.8	+19.0	+11.4; +27.1
Age adjusted	−40.9	−55.9; −20.9	−11.3	−21.3; −0.2	+12.9	−4.4; +33.3	+14.2	+6.9; +22.1

EPC=estimated percentage change; 95% CI=95% confidence intervals; ES area=early screening area; LS area=late screening area

). In the period 1985–2000, the overall age-standardised mortality decreased by 40.9% in the ES area (*P*<0.001), and by 11.3% in the LS area (*P*=0.030). Significant decreases in the ES area were detected in age groups 45–54 years (61.1%; *P*=0.018) and 65–74 years (44.7%; *P*=0.049). In the 75–84 age group, the decrement was 45.2% (*P*=0.053). In the LS area, no significant decreases were detected in any age group.

In the period 1985–1999, the overall age-standardised incidence increased by 12.9% in the ES area (*P*=0.190), and by 14.2% in the LS area (*P*<0.001) (Table 1). In the ES area, no significant increases or decreases were detected in any age groups; in the LS area, where the prevalence screening was carried out in the period 1990–1994, significant increases were detected in groups aged 55–64 years (21.0%; *P*=0.009) and 85 years and over (45.1%; *P*=0.012).

## DISCUSSION

Decreases in breast cancer mortality have been variously reported following the introduction of mammographic screening on a regional level (Tornberg *et al*, 1994; Quinn and Allen, 1995; Garne *et al*, 1997; Barchielli and Paci, 2001; Broeders *et al*, 2001; Tabar *et al*, 2001; Duffy *et al*, 2002). Randomised prospective trials indicate that benefits in terms of cumulative breast cancer mortality start to emerge 4–10 years after randomisation (Nyström *et al*, 2002). We observed a similar trend in the mortality rates in the two areas up to 1993, although the mortality rates in the ES area were lower than those in the LS area. Afterwards a marked fall occurred in ES area, whereas only a slight decrease was observed in the LS area. The relatively low compliance in the first years of the programme in both areas (about 55%) and the long enrolment period (on average 2.5 years) in the LS area (Paci *et al*, 2002a, 2002b) could have diluted the effect so that a decrease in mortality rates was observed only after a longer interval.

Our findings suggest that the drop in mortality in the ES area (41%) could be explained by both service screening and better care. The slight decrease in mortality in the LS area (11%) could be explained by better care, while service screening of the period 1990–2000 in the LS area could have been responsible for only a small number of deaths occurring in the target population. In fact, it has been estimated that in the city of Florence, the breast cancer mortality reduction at ages 50–74 years attributable to screening was 3.2% over the period 1990–1999 (Paci *et al*, 2002a). Thus, allowing a 11% reduction due to better care, a reduction of about 30% (41–11%) is attributable to screening in the ES area over the period 1985–2000. These findings are consistent with the mortality reduction of about 25% in screened populations shown by randomised trials (Vainio and Bianchini, 2002).

The decrease in mortality at ages 45–54 years in the ES area could be related to the involvement in mammographic screening of women aged 40–49 years in the ES area until 1993. In 1985–1993, 33% of mammograms in the ES area (about 2700 per year) were performed on women aged 40–49 years. No mortality reduction would be expected from screening in the younger age group (35–44 years) or in the 85 years and over age group.

In evaluating these results, it must be borne in mind that the ES area, where the incidence rates were lower, was a less urbanised area during the period of observation. The significant increase in incidence recorded only in the LS area (of 14.2%), particularly in women aged 55–64 years (21.0%), could be explained by earlier detection. Increases in incidence have been recorded in other Western countries in relation to screening (Quinn and Allen, 1995; Garne *et al*, 1997; Persson *et al*, 1998; Chu *et al*, 1996; Rostgaard *et al*, 2001). In the ES area, no significant increase in incidence was detected. It is likely that an increase in incidence in the ES area should have occurred in the 1970s, as a result of screening.

Incidence trends in the ES and LS areas do not seem to explain the observed differences in mortality rates during the study period. The present results seem to confirm that service screening can reduce breast cancer mortality rates.
